# Characterizing patterns of opioid and stimulant use by route and associations with non-fatal overdose and xylazine use in people who have injected drugs from Baltimore, MD, 2023–2024

**DOI:** 10.1016/j.dadr.2025.100403

**Published:** 2025-12-06

**Authors:** Jacqueline E. Rudolph, Liz M. Martinez-Ocasio, Kenneth A. Feder, Catherine Tomko, Javier A. Cepeda, Gregory D. Kirk, Shruti H. Mehta, Danielle German, Becky L. Genberg

**Affiliations:** a Department of Epidemiology, Bloomberg School of Public Health, Johns Hopkins University, Baltimore, MD, USA; b Department of Mental Health, Bloomberg School of Public Health, Johns Hopkins University, Baltimore, MD, USA; c Department of Health, Behavior & Society, Bloomberg School of Public Health, Johns Hopkins University, Baltimore, MD, USA; d Division of Infectious Diseases, Johns Hopkins School of Medicine, Baltimore, MD, USA

**Keywords:** Opioid, Fentanyl, Stimulant, Cocaine, Injection, Non-fatal overdose, Xylazine

## Abstract

**Background:**

The fourth wave of the overdose epidemic in the United States (US) is characterized by deaths involving fentanyl with stimulants. Understanding patterns of substance use as novel drugs continue to emerge is critical to inform overdose prevention. Among people with a history of injection drug use in Baltimore, MD, we examined opioid-stimulant use by route and the association of injection with non-fatal overdose and xylazine use.

**Methods:**

We included 1132 participants in the AIDS Linked to the IntraVenous Experience cohort with a study visit in 2023–2024. We characterized participants by self-reported past-six-month use of any opioid and any stimulant by route using descriptive statistics. Adjusted associations were estimated using targeted minimum loss-based estimation.

**Results:**

Most participants were Black (68 %), male (67 %), and older (median: 56 years). 53 % reported using both opioids and stimulants; these participants were more likely to report using other substances, non-fatal overdose, depressive symptoms, and homelessness. Among participants using both, those injecting both were more likely to report non-fatal overdose and xylazine use than those using by non-injection (overdose: PD = 22.3 %, 95 % CI= 17.2 %-27.5 %; xylazine: PD = 13.3 %, 95 % CI = 4.7 %-21.9 %).

**Conclusions:**

We provide one of the most comprehensive summaries of substance use during the fourth wave in Baltimore, a city with the highest rate of fatal overdose in the US during the study period. Use of multiple substances was common, and many participants reported injection. Overdose prevention should be targeted to those injecting both drugs as this pattern had heightened overdose risk.

## Highlights

• Over half of sample reported using both opioids and stimulants in past 6 months.
• Use of both related to use of other drugs, social disadvantage, and depression.
• Xylazine use found predominantly in participants reporting use of both.
• Among those reporting both, injecting both associated with non-fatal overdose.

## 1 Introduction

Globally, one in five people who inject drugs (PWID) have experienced a non-fatal overdose in the past year, and fatal overdose remains the top cause of death among PWID (Colledge et al., 2019, Mathers et al., 2013). Over the past decade in the United States (US), there has been an exponential increase in overdose deaths involving the synthetic opioid fentanyl, with many overdoses occurring in individuals using fentanyl in combination with stimulants, predominantly cocaine or methamphetamine, representing a behavioral shift from use of opioids alone to polysubstance use (Ciccarone, 2021, Compton et al., 2021, Friedman and Shover, 2023, Townsend et al., 2022).

Use of multiple substances, including opioids and stimulants, is indeed the norm among people with substance use disorders (Cicero et al., 2020, Karamouzian et al., 2022, Leri et al., 2003, Rockhill et al., 2025). In Baltimore, Maryland, the setting of this study, heroin and cocaine (both powder and crack) have been central to the illicit drug markets since the 1980s, and Baltimore PWID have long used the two drugs either simultaneously (as speedball) or sequentially (Feder et al., 2023, Genberg et al., 2011, Vlahov et al., 1991). Since the emergence of fentanyl in late 2013, the Baltimore drug market has shifted nearly universally to synthetic opioids (Russell et al., 2023), and the city has seen a similar exponential increase in overdose deaths as other parts of the US (Maryland Department of Health, 2025). Of note, as of 2025, Baltimore City had the highest rate of fatal overdose of all US counties (San Francisco Chronicle, 2025).

There are hypothesized supply and demand explanations for the rise in overdoses involving fentanyl and stimulants in the US. Regarding demand, people who use drugs (PWUD) may use opioids and stimulants in combination to experience the effects of both drugs simultaneously, to enhance or counteract the impact of one substance with another, or to mitigate symptoms of opioid withdrawal (Boileau-Falardeau et al., 2022, Compton et al., 2021, Crummy et al., 2020, Leri et al., 2003). These motivations were reported when heroin was the primary opioid in drug markets; the need to counterbalance the effects of opioids may be even more relevant with respect to fentanyl, given the higher potency and shorter half-life compared with heroin (Ciccarone, 2017, Friedman and Shover, 2023). Regarding supply, there is some evidence of limited cross-contamination of the stimulant supply with fentanyl, leading to unintentional co-use (Glidden et al., 2023, Russell et al., 2023, Wagner et al., 2023). Drug contamination increases risk of overdose, particularly among those with little to no tolerance to the unintended substance (Glidden et al., 2023, Mariano and Berk, 2024, McCall Jones et al., 2017). In this paper, we focus on intentional use.

The US overdose epidemic was further complicated by the emergence of xylazine as an adulterant of illicitly manufactured fentanyl. Xylazine, which has been present in the Puerto Rican drug supply for decades (Reyes et al., 2012, Torruella, 2011), began to appear with more frequency in the Northeast US drug supply in the late 2010s and by 2022 had become pervasive in a growing proportion of the US (Cano et al., 2024, D’Orazio et al., 2023, Johnson et al., 2021, Kariisa et al., 2023, Russell et al., 2023, U.S. Department of Justice Drug Enforcement Administration, 2022). While xylazine may enhance and prolong the effects of fentanyl, initial evidence suggests PWUD have mixed opinions on the effects of using xylazine and concerns about the harms associated with its use (Friedman et al., 2022, Heidari et al., 2025, Hochheimer et al., 2024, Reed et al., 2022). Xylazine may increase risk of fatal overdose because it has synergistic pharmacological effects with fentanyl and because it is non-responsive to naloxone (Friedman et al., 2022, Nunez et al., 2021). Additionally, xylazine is strongly linked to severe skin wounds and lesions, even in areas distal from the injection site (Friedman et al., 2022, German et al., 2024, Quijano et al., 2023, Reyes et al., 2012, Torruella, 2011). Nevertheless, individuals who use fentanyl may have limited options given the constraints of illicit drug markets and may use xylazine despite preferences to avoid it. While post-mortem data suggests nearly all individuals using xylazine at time of overdose were using fentanyl and a meaningful proportion were also using stimulants (Bradford et al., 2024, Johnson et al., 2021, Kariisa et al., 2023, Vohra et al., 2023), there remains limited epidemiological data in the community setting characterizing xylazine use and how it overlaps with patterns of polysubstance use (German et al., 2024, Khezri et al., 2025, Shrestha et al., 2025).

Here, we characterized patterns of opioid and stimulant use in the AIDS Linked to the Intravenous Experience (ALIVE) study: a community-based cohort in Baltimore, Maryland. First, we described patterns of opioid and stimulant use, stratified by whether participants injected those drugs. Second, we examined how participant characteristics and use of other classes of harmful substances (illicit and legal) differed across opioid-stimulant use patterns. Third, among participants using both opioids and stimulants, we examined whether injecting both substances, relative to injecting one or using by non-injection, was associated with three adverse outcomes: non-fatal overdose, xylazine use, and high-frequency injection.

Investigating the evolving epidemiology of using opioids and stimulants is critical, given the increase in this use pattern across the US and the literature reporting associations between polysubstance use (defined heterogeneously across studies) with higher rates of nonfatal overdose (Gicquelais et al., 2019, Karamouzian et al., 2022, Schneider et al., 2019), drug use practices that increase risk for infection (Gicquelais et al., 2019, Karamouzian et al., 2022, Leeman et al., 2016), poorer mental health (Betts et al., 2016, DiGuiseppi et al., 2020, Karamouzian et al., 2022, Leeman et al., 2016), homelessness (DiGuiseppi et al., 2020, Karamouzian et al., 2022, Leeman et al., 2016, Schneider et al., 2019), unemployment (Karamouzian et al., 2022, Leeman et al., 2016), interactions with the criminal justice system (Betts et al., 2016, Karamouzian et al., 2022, Leeman et al., 2016), and reduced drug treatment effectiveness (Leri et al., 2003). Furthermore, our findings are an important update to work conducted prior to the COVID-19 pandemic (Rudolph et al., 2024). As evidenced by a mass overdose event in July 2025, the overdose epidemic in Baltimore remains unpredictable (Cohn et al., 2025). Understanding substance use patterns in a setting like Baltimore, where emerging adulterants are present, can inform our understanding of overdose prevention as drug markets across the globe continue to evolve (Meyer et al., 2023).

## 2 Methods

### 2.1 Study sample

We used data from the ALIVE Study, a community-based cohort of adults who have injected drugs living in or near Baltimore. Since the study’s inception in 1988, there have been five additional recruitment waves: 1994–1995, 1998, 2005–2008, 2015–2018, and 2023-present. Details on the study design can be found elsewhere (Vlahov et al., 1991); we provide a brief summary in the Supplement. Participants make twice-annual visits to a study clinic where they complete standardized surveys on substance use, related behaviors, and health outcomes and provide a blood sample for communicable disease and other testing. The Johns Hopkins University institutional review board approved the study; written informed consent was obtained at time of enrollment.

In this analysis, we included each participant’s first visit between January 1, 2023 (the first date of the most recent recruitment wave) and December 31, 2024. We excluded 2 participants for having missing data on route of opioid use (specifically, missingness in survey items assessing oral and snorted fentanyl), yielding an analytic sample of 1132 participants.

### 2.2 Measures

At each study visit, participants were asked whether they used cocaine, heroin, speedball, fentanyl, crystal methamphetamine, xylazine, hallucinogens, methadone, buprenorphine, prescription painkillers, prescription sedatives, prescription tranquilizers, and prescription stimulants in the past 6 months. For all prescription drugs (including methadone and buprenorphine), participants were asked whether they used the drugs in “any way that was not prescribed.” Hereafter, “use” of these substances will refer to “non-medical use.” In this analysis, we combined heroin/fentanyl into one category because, during the study period, nearly all substances marketed as “heroin” in Baltimore were fentanyl and referring to the drug as heroin likely reflected naming preferences rather than the substance used (Russell et al., 2023). For each substance, participants were asked the route by which they used the substance: injection, smoking, snorting, or oral.

**Fig. 1:**
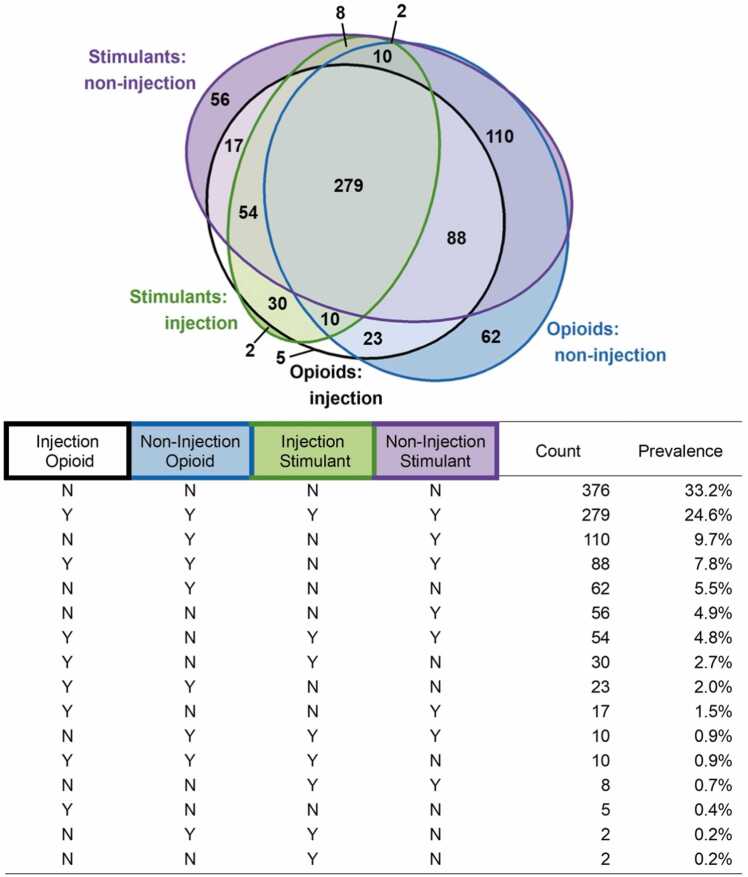
Combinations of opioid and stimulant use by route.

Our main outcomes were self-report of any opioid use and any stimulant use in the past 6 months. We defined “any opioid use” as use of heroin/fentanyl, speedball, methadone, buprenorphine, or prescription painkillers. We defined “any stimulant use” as use of cocaine, speedball, crystal methamphetamine, or prescription stimulants. We classified use of opioids and stimulants based on whether the participant used each substance by injection or non-injection (smoking, snorting, or orally ingesting). This resulted in 16 mutually exclusive categories describing opioid-stimulant use by route (Fig. 1).

To describe our study sample, we examined a number of variables. Variables measured at study baseline included sex (male, female), race/ethnicity (non-Hispanic Black, non-Hispanic white, Hispanic, other), education (less than high school, high school or greater), age at first injection, and enrollment cohort (pre-2005, 2005, 2015, 2023). Variables measured at the study visit included age, marital status (never, ever married), HIV status (positive/negative), HCV antibody status (positive/negative), residential location (within or outside Baltimore City based on geocoding of self-reported address), formal employment, homelessness or housing instability, incarceration greater than 7 days, frequency of injection (none, less than daily, daily for all substances and for heroin, fentanyl, cocaine, methamphetamine, and speedball), frequency of non-injection drug use (same categories), any MOUD (methadone, buprenorphine, or naltrexone), any drug support group, non-fatal overdose, depressive symptoms (based on a score of 23 or higher on the CESD), cigarette use, alcohol use (none, moderate, or heavy based on the AUDIT), and cannabis use. With the exception of age, HIV, and HCV, all time-updated variables reflect self-report of the last 6 months. HIV status was based on detection of antibodies to HIV, using enzyme-linked immunosorbent assay with Western blot confirmation. All participants were tested for HCV antibodies at cohort entry, and repository specimens are routinely tested for HCV seroconversion.

### 2.3 Statistical analysis

To characterize the diversity of opioid-stimulant use by route, we ranked the 16 categories of opioid-stimulant use by route by number of participants in each category. We visualized overlap in the categories using Euler plots, a generalization of Venn diagrams that accommodates multiple overlapping ellipses with the area of overlap approximately reflecting the prevalence of the overlap within the sample.

To compare participant characteristics across opioid-stimulant categories, we generated descriptive statistics for each variable listed in Measures, overall and stratified by membership in the 6 most common categories. In a sensitivity analysis, we compared characteristics by enrollment cohort to assess the extent to which the observed trends by opioid-stimulant use were driven by cohort effects.

Among those reporting use of both opioids and stimulants, we classified participants into three mutually-exclusive groups: used by non-injection routes, injected only opioids or only stimulants (hereafter, “injected one drug”), and injected both drugs. We examined the association between route category and three self-reported outcomes: non-fatal overdose, xylazine use, and daily injection in the past 6 months. In the analysis of daily injection, we excluded those using by non-injection only. Specifically, we compared the prevalence of each outcome across exposure categories using prevalence differences (PD). We estimated the PDs using targeted minimum loss-based estimation (TMLE), controlling for age, sex, race (binary Black/other), and enrollment cohort. TMLE is a modern approach that leverages the results of multiple models (specifically, a propensity score model and an outcome model) for more robust inference (Van Der Laan and Rubin, 2006, Williams and Díaz, 2023). TMLE also allows us to use machine learning to fit those models, thereby mitigating biases that can arise when the strict assumptions made by traditional regression models regarding the form of the data (e.g., the outcome distribution or a linear relationship between the independent and dependent variables) are wrong (Rudolph et al., 2018). We fit each model using the ensemble algorithm SuperLearner, which synthesizes results across several algorithms (here, we used the mean, generalized linear models with main terms and interaction terms, random forest, and neural networks) (Van der Laan et al., 2007). We obtained 95 % confidence intervals (CI) using the variance of the efficient influence function.

There was minimal missingness (<1 % of sample) in any measure examined in this analysis. Below, the reported prevalence of participant characteristics other than city residence reflects the prevalence among those with non-missing status. For city residence, we report the prevalence of those without valid geocoding, a meaningful category reflecting those with no address to report on the visit date. When assessing the association between injection and non-fatal overdose and self-reported xylazine use, we excluded 3 and 7 participants with missing outcome data, respectively.

## 3 Results

### 3.1 Description of study sample

Of the 1132 participants in the analysis (Table 1), the majority were Black (68 %), male (67 %), and living within Baltimore City (77 %). Almost half (42 %) enrolled in 2023–2024.The cohort was highly socioeconomically disadvantaged, with only 10 % actively employed and 31 % having recently experienced homelessness.

**Table 1:** Participant characteristics and substance use behaviors, overall and within the 6 most common opioid-stimulant use patterns.

Variable	Overall	No Opioids; No Stimulants	Inj. & Non-Inj. Opioids; Inj. & Non-Inj. Stimulants	Non-Inj. Opioids; Non-Inj. Stimulants	Inj. & Non-Inj. Opioids; Non-Inj. Stimulants	Non-Inj. Opioids	Non-Inj. Stimulants
No. participants	1132	376	279	110	88	62	56
Participant Characteristics							
Age at visit, median (IQR)	56 (44, 63)	63 (58, 68)	45 (38, 54)	55 (51, 62)	45 (38, 58)	58 (54, 64)	59 (53, 65)
Age first injected, med. (IQR)	18 (14, 23)	20 (16, 26)	14 (12, 17)	21 (16, 25)	15 (12, 19)	21 (17, 25)	21 (17, 26)
Enrollment cohort							
Pre-2005	263 (23 %)	189 (50 %)	6 (2 %)	14 (13 %)	1 (1 %)	19 (31 %)	19 (34 %)
2005	173 (15 %)	104 (28 %)	3 (1 %)	22 (20 %)	10 (11 %)	10 (16 %)	10 (18 %)
2015	224 (20 %)	66 (18 %)	12 (4 %)	50 (45 %)	9 (10 %)	27 (44 %)	19 (34 %)
2023	472 (42 %)	17 (5 %)	258 (92 %)	24 (22 %)	68 (77 %)	6 (10 %)	8 (14 %)
Female sex	377 (33 %)	118 (31 %)	93 (33 %)	44 (40 %)	34 (39 %)	18 (29 %)	28 (50 %)
Race/ethnicity							
Non-Hispanic Black	760 (67 %)	341 (91 %)	135 (48 %)	76 (69 %)	40 (45 %)	52 (84 %)	43 (77 %)
Non-Hispanic white	306 (27 %)	19 (5 %)	123 (44 %)	28 (25 %)	43 (49 %)	4 (6 %)	11 (20 %)
Hispanic	9 (1 %)	6 (2 %)	0 (0 %)	1 (1 %)	1 (1 %)	1 (2 %)	0 (0 %)
Other	57 (5 %)	10 (3 %)	21 (2 %)	5 (5 %)	4 (5 %)	5 (8 %)	2 (4 %)
Never married	622 (55 %)	198 (53 %)	162 (58 %)	59 (54 %)	52 (60 %)	29 (47 %)	30 (54 %)
Less than high school	537 (47 %)	207 (55 %)	97 (35 %)	56 (51 %)	37 (42 %)	34 (55 %)	32 (57 %)
Employed	115 (10 %)	72 (19 %)	10 (4 %)	5 (5 %)	5 (6 %)	6 (10 %)	8 (14 %)
Any homelessness	354 (31 %)	12 (3 %)	178 (64 %)	29 (26 %)	49 (56 %)	6 (10 %)	7 (12 %)
Incarcerated ≥ 1 week	40 (4 %)	3 (1 %)	13 (5 %)	5 (5 %)	5 (6 %)	0 (0 %)	0 (0 %)
Residence							
In City	872 (77 %)	326 (87 %)	190 (68 %)	94 (85 %)	58 (66 %)	52 (84 %)	44 (79 %)
Outside City	177 (16 %)	39 (10 %)	52 (19 %)	13 (12 %)	20 (23 %)	10 (16 %)	9 (16 %)
No geocoding	83 (7 %)	11 (3 %)	37 (13 %)	3 (3 %)	10 (11 %)	0 (0 %)	3 (5 %)
Depressive symptoms	374 (33 %)	42 (11 %)	153 (55 %)	47 (43 %)	38 (43 %)	17 (27 %)	17 (30 %)
HIV-positive	222 (20 %)	137 (36 %)	12 (4 %)	20 (18 %)	6 (7 %)	10 (16 %)	22 (39 %)
HCV-positive	775 (71 %)	298 (80 %)	158 (61 %)	75 (70 %)	42 (51 %)	41 (66 %)	46 (82 %)
Any MOUD	559 (49 %)	139 (37 %)	163 (58 %)	61 (55 %)	49 (56 %)	30 (48 %)	27 (48 %)
Drug support group	385 (34 %)	117 (31 %)	109 (39 %)	40 (36 %)	29 (33 %)	25 (40 %)	13 (23 %)
Substance Use							
Cigarettes	916 (81 %)	233 (62 %)	259 (93 %)	96 (87 %)	84 (95 %)	54 (89 %)	45 (80 %)
Alcohol							
None	661 (58 %)	255 (68 %)	148 (53 %)	64 (58 %)	45 (51 %)	33 (53 %)	21 (37 %)
Moderate	231 (20 %)	60 (16 %)	58 (21 %)	21 (19 %)	23 (26 %)	12 (19 %)	18 (32 %)
Heavy	240 (21 %)	61 (16 %)	73 (26 %)	25 (23 %)	20 (23 %)	17 (27 %)	17 (30 %)
Cannabis	520 (46 %)	80 (21 %)	207 (74 %)	56 (51 %)	51 (58 %)	23 (37 %)	26 (46 %)
Cocaine	656 (58 %)	0 (0 %)	279 (100 %)	109 (99 %)	88 (100 %)	0 (0 %)	55 (98 %)
Heroin/fentanyl	667 (59 %)	0 (0 %)	277 (100 %)	108 (98 %)	88 (100 %)	49 (79 %)	0 (0 %)
Speedball	298 (26 %)	0 (0 %)	229 (82 %)	0 (0 %)	0 (0 %)	0 (0 %)	0 (0 %)
Methamphetamine	61 (5 %)	0 (0 %)	41 (15 %)	2 (2 %)	4 (5 %)	0 (0 %)	2 (4 %)
Xylazine	134 (12 %)	0 (0 %)	85 (31 %)	4 (4 %)	14 (16 %)	0 (0 %)	0 (0 %)
Hallucinogen	45 (4 %)	0 (0 %)	28 (10 %)	3 (3 %)	6 (7 %)	1 (2 %)	1 (2 %)
Methadone	134 (12 %)	0 (0 %)	89 (32 %)	10 (9 %)	15 (17 %)	9 (15 %)	0 (0 %)
Buprenorphine	62 (5 %)	0 (0 %)	47 (17 %)	2 (2 %)	6 (7 %)	2 (3 %)	0 (0 %)
Rx painkillers	86 (8 %)	0 (0 %)	58 (21 %)	3 (3 %)	9 (10 %)	9 (15 %)	0 (0 %)
Rx sedatives	62 (5 %)	0 (0 %)	43 (15 %)	3 (3 %)	7 (8 %)	1 (2 %)	1 (2 %)
Rx tranquilizer	105 (9 %)	1 (<1 %)	69 (25 %)	9 (8 %)	10 (11 %)	2 (3 %)	0 (0 %)
Rx stimulants	56 (5 %)	0 (0 %)	42 (15 %)	2 (2 %)	8 (9 %)	0 (0 %)	1 (2 %)
No. illicit substances							
0	374 (33 %)	374 (99 %)	0 (0 %)	0 (0 %)	0 (0 %)	0 (0 %)	0 (0 %)
1	134 (12 %)	2 (1 %)	0 (0 %)	0 (0 %)	0 (0 %)	55 (89 %)	53 (95 %)
2	219 (19 %)	0 (0 %)	23 (8 %)	93 (85 %)	48 (55 %)	4 (6 %)	2 (4 %)
3	166 (15 %)	0 (0 %)	79 (28 %)	8 (7 %)	19 (22 %)	2 (3 %)	1 (8 %)
4	95 (8 %)	0 (0 %)	60 (22 %)	4 (4 %)	10 (11 %)	1 (2 %)	0 (0 %)
5	60 (5 %)	0 (0 %)	45 (16 %)	1 (1 %)	8 (9 %)	0 (0 %)	0 (0 %)
6 +	84 (7 %)	0 (0 %)	72 (26 %)	4 (4 %)	3 (3 %)	0 (0 %)	0 (0 %)
Injection frequency^a^							
None	608 (54 %)	376 (100 %)	0 (0 %)	108 (98 %)	4 (5 %)	62 (100 %)	56 (100 %)
Less than daily	171 (15 %)	0 (0 %)	77 (28 %)	0 (0 %)	32 (36 %)	0 (0 %)	0 (0 %)
Daily	353 (31 %)	0 (0 %)	202 (72 %)	2 (2 %)	52 (59 %)	0 (0 %)	0 (0 %)
Non-injection frequency							
None	382 (34 %)	332 (88 %)	1 (<1 %)	0 (0 %)	1 (1 %)	6 (10 %)	1 (2 %)
Less than daily	245 (22 %)	27 (7 %)	52 (19 %)	44 (40 %)	19 (22 %)	32 (52 %)	31 (55 %)
Daily	504 (45 %)	17 (5 %)	226 (81 %)	66 (60 %)	68 (77 %)	24 (39 %)	24 (43 %)
Non-fatal overdose	129 (11 %)	0 (0 %)	77 (28 %)	6 (5 %)	15 (17 %)	3 (5 %)	2 (4 %)

Abbreviations: HCV, hepatitis C virus; HIV, human immunodeficiency virus; Inj., injection; IQR, interquartile range; med., median; MOUD, medication for opioid use disorder; No., number of; Non-Inj., non-injection; Rx, non-medical use of prescription drug

^a^Frequency of injection is based on self-reported number of times participant injected any drug in the last 6 months and could differ from the responses related to injection of specific substances. There were 4 individuals classified as not injecting based on this question who reported injecting heroin (2 reporting daily, 1 reporting <1 per month) or fentanyl (1 reporting 1–3 per month).

### 3.2 Patterns of opioid-stimulant use

Fig. 1 illustrates the overlap in opioid-stimulant use by route. One third of participants (n = 376) reported using neither opioids nor stimulants. Of the 756 (67 %) participants reporting use of either stimulants or opioids, 600 (79 %) reported using both. The 5 most prevalent categories were: (1) 279 (25 %) participants used injection and non-injection opioids and injection and non-injection stimulants; (2) 110 (10 %) participants used non-injection opioids and non-injection stimulants; (3) 88 (8 %) participants used injection and non-injection opioids and non-injection stimulants; (4) 62 (5 %) participants used non-injection opioids; and (5) 56 participants used non-injection stimulants.

### 3.3 Participant characteristics by opioid-stimulant use

A comparison of all drug use behaviors and other participant characteristics by the common patterns of opioid-stimulant use are provided in Table 1. Below, we summarize several notable trends. First, participants using neither opioids nor stimulants rarely used other illicit drugs. They were also less likely to report using cigarettes, alcohol, or cannabis relative to participants using either opioids or stimulants. Second, participants who reported injecting either opioids or stimulants were more likely than other participants to be using other substances. Participants who used injection and non-injection opioids and injection and non-injection stimulants had the highest prevalence of non-medical use of prescription drugs (ranging from 15 % for stimulants and sedatives to 32 % for methadone), methamphetamine (15 %), xylazine (31 %), and cannabis (74 %). Only 8 % of these participants reported using only opioids and stimulants; 26 % used 6 or more illicit substances. Third, reporting use of both opioids and stimulants, especially by injection, was associated with drug-related harms, social disadvantage, and other health concerns. Nearly all non-fatal overdoses were observed among individuals using both opioids and stimulants. Moreover, 64 % of those using injection and non-injection opioids and injection and non-injection stimulants, as well as 55 % of those using injection and non-injection opioids and non-injection stimulants, experienced homelessness in the past 6 months. Finally, most participants using both stimulants and opioids (particularly those injecting either) enrolled in the cohort in 2023–2024, and many of the characteristics that differed by opioid-stimulant use also differed by enrollment cohort (Supplementary Table 1).

### 3.4 Association of injection with adverse outcomes

**Fig. 2:**
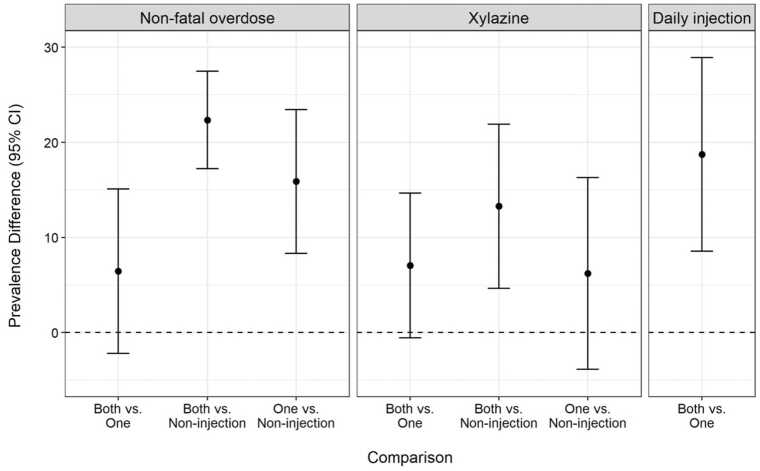
Association between injection and non-fatal overdose, xylazine use, and daily injection among participants reporting use of both opioids and stimulants in the last 6 months, adjusting for age, sex, race, and enrollment cohort.

Among the 600 participants reporting use of both opioids and stimulants, 110 (18 %) used by non-injection, 117 (20 %) injected one drug (with 90 % injecting opioids only), and 373 (62 %) injected both drugs. Of those who injected both drugs, 26 % reported a non-fatal overdose and 29 % reported using xylazine. This was higher than the 19 % reporting non-fatal overdose and 18 % reporting xylazine use among those injecting one drug and the 5 % reporting non-fatal overdose and 4 % reporting xylazine use among those using by non-injection. Participants injecting both drugs were more likely to report daily injection than those injecting one drug (Table 2), generally and across all specific substances except methamphetamine (for which they were more likely to report any injection). The adjusted prevalence differences were similar to the crude differences (Fig. 2). Participants injecting both drugs had a prevalence of non-fatal overdose that was 6.5 % (95 % CI: −2.2 %, 15.1 %) higher than those injecting one drug and 22.3 % (95 % CI 17.2 %, 27.5 %) higher than those using by non-injection. Participants injecting both drugs had a prevalence of self-reported xylazine use that was 7.1 % (95 % CI: −0.5 %, 14.7 %) higher than those injecting one drug and 13.3 % (95 % CI: 4.7 %, 21.9 %) higher than those using by non-injection. Differences were closer to the null when comparing participants who injected one drug against those using by non-injection (non-fatal overdose: 15.9 % [95 % CI 8.3 %, 23.4 %]; xylazine: 6.2 %, [95 % CI: −3.9 %, 16.3 %]). Participants injecting both drugs were more likely to report daily injection (PD: 18.7 % [95 % CI: 8.6 %, 28.9 %]) than those injecting one drug.

**Table 2:** Among participants who used both opioids and stimulants, comparison of the prevalence of non-fatal overdose, xylazine use, and injection frequency (overall and by substance) between participants who injected both, who injected either opioids or stimulants (injected one), and those who used but did not inject either opioids or stimulants.

Variable	Injected Both^a^ (n = 373)	Injected One^b^ (n = 117)	Non-Injection Only (n = 110)
Non-fatal overdose	95 (26 %)	22 (19 %)	6 (5 %)
Xylazine use	106 (29 %)	21 (18 %)	4 (4 %)
Any injection			
None	0 (0 %)	5 (4 %)	NA
Less than daily	64 (26 %)	48 (41 %)	NA
Daily	277 (74 %)	64 (55 %)	NA
Heroin injection			
None	26 (7 %)	28 (24 %)	NA
Less than daily	181 (49 %)	47 (40 %)	NA
Daily	166 (44 %)	42 (36 %)	NA
Fentanyl injection			
None	94 (25 %)	53 (45 %)	NA
Less than daily	147 (39 %)	37 (32 %)	NA
Daily	132 (35 %)	27 (23 %)	NA
Cocaine injection			
None	44 (12 %)	105 (90 %)	NA
Less than daily	274 (73 %)	11 (9 %)	NA
Daily	55 (15 %)	1 (1 %)	NA
Methamphetamine injection			
None	338 (91 %)	115 (98 %)	NA
Less than daily	33 (9 %)	1 (1 %)	NA
Daily	2 (1 %)	1 (1 %)	NA
Speedball injection			
None	75 (20 %)	117 (100 %)	NA
Less than daily	222 (60 %)	0 (0 %)	NA
Daily	76 (20 %)	0 (0 %)	NA

Abbreviations: n, number of participants

^a^Category includes the 279 who used injection and non-injection opioids and injection and non-injection stimulants; the 54 who used injection opioids and injection and non-injection stimulants; the 30 who used injection opioids and injection stimulants; and the 10 who used injection and non-injection opioids and injection stimulants

^b^Category includes the 88 who used injection and non-injection opioids and non-injection stimulants; the 17 who used injection opioids and non-injection stimulants; the 10 who used non-injection opioids and injection and non-injection stimulants; and the 2 who used non-injection opioids and injection stimulants

## 4 Discussion

In this sample of adults who have injected drugs, we found heterogeneous patterns of self-reported opioid-stimulant use (which in this setting is predominantly heroin/fentanyl-cocaine use) that were related to the use of other illicit and legal drugs, social disadvantage, and depression. Reporting use of both opioids and stimulants, especially injection of both, was associated with the use of xylazine and non-fatal overdose. Among participants using both substances, daily injection was more common among those injecting both drugs, relative to those injecting only one. While the association between using both opioids and stimulants and nonfatal overdose, depression, and homelessness have previously been reported (Cicero et al., 2020, Compton et al., 2021, Leeman et al., 2016, Rudolph et al., 2024, Timko et al., 2018), our data provide one of the most comprehensive summaries of substance use among people who have injected drugs in Baltimore following the introduction of synthetic opioids and xylazine into drug markets and reveal several novel findings related to the relationship between injection of both opioids and stimulants and self-reported non-fatal overdose, high frequency injection, and xylazine use.

Prevalence of non-fatal overdose was higher among participants reporting use of both opioids and stimulants than among participants using either stimulants or opioids. Use of multiple substances and use by injection are independently risk factors for overdose (Darke and Hall, 2003); we sought to assess whether, among participants using both opioids and stimulants, injection of more substances was associated with non-fatal overdose. Our data were largely consistent with an increasing prevalence of non-fatal overdose with increasing number of substances injected. However, the difference in non-fatal overdose between those injecting both and injecting one was half the difference seen between those injecting one and those using by non-injection routes – despite the fact that those who injected both drugs were more likely to report daily injection and were the only group for whom we knew participants used opioids and stimulants concurrently (having a high prevalence of speedball use). This may suggest that, among individuals reporting use of opioids and stimulants, a larger impact on non-fatal overdose would be seen through injection cessation rather than limiting frequency of injection or number of substances injected. Further research examining prospective outcomes is needed to assess this hypothesis.

The emergence of xylazine in the Baltimore fentanyl supply is apparent in these data. Xylazine use was exclusively reported among individuals using both opioids and stimulants, with nearly a third of those in the injection and non-injection opioid and injection and non-injection stimulant category reporting xylazine. Prior studies in the US examining xylazine in decedents have universally found that xylazine is used with fentanyl and that overlap with stimulants and benzodiazepines is also common (Bradford et al., 2024, Johnson et al., 2021, Kariisa et al., 2023, Vohra et al., 2023). Here, self-reported xylazine use was most prevalent in people reporting use of both opioids and stimulants (with modest prevalence in those using injection and non-injection opioids) and was almost exclusively seen in participants injecting one or both substances. This may reflect genuine xylazine use patterns among people who have injected drugs in Baltimore, but it may also reflect that those injecting both opioids and stimulants are more aware of the presence of xylazine in the fentanyl supply, since we measure known use. Research among PWUD in the US has shown that awareness of xylazine remains low (Hochheimer et al., 2024, Quijano et al., 2023, Shrestha et al., 2025). This implies that the true prevalence of xylazine use may be higher in this sample than reflected in the data.

There were limitations of this study. This analysis was cross-sectional, representing a snapshot of substance use in ALIVE between 2023 and 2024. All estimated associations were descriptive. With sufficient follow-up, we will examine trends longitudinally and consider outcomes like fatal overdose prospectively. The estimated associations and their standard errors also did not account for the possibility of clustering of participants caused by the study referral process or social networks. Furthermore, all substance use variables (type, route, and frequency) were based on self-report of the past 6 months. In addition to the possibility of misclassification due to social desirability bias and recall bias, this limits our ability to assess use of multiple substances on a more granular scale. With the exception of speedball, we cannot say whether the reported opioids and stimulants were used in combination.

Finally, the ALIVE cohort may not be generalizable to other communities of people who have injected drugs. The cohort is older, majority Black, urban, and predominately uses heroin/fentanyl and cocaine. While many participants who enrolled prior to the COVID-19 pandemic transitioned to not using or to using via non-injection routes (Feder et al., 2023, Genberg et al., 2011, Shah et al., 2006), the most recent enrollees used many different substances (even drugs, such as methamphetamine, that have been uncommon in this cohort), engaged in high frequency injection, and experienced other factors associated with overdose (e.g., 60 % reporting any homelessness and 50 % reporting depressive symptoms). This reflects how the cohort intentionally replenishes from the community of people who have injected drugs in Baltimore. While recruitment practices have changed little over time, cohort composition has (Cepeda et al., 2019), and these shifts in characteristics reveal shifts in our underlying target population and in drug trafficking and social patterns across the Northeast US (Friedman and Shover, 2023, Streck et al., 2023, Wakeman et al., 2021). These participants are at greatest risk for non-fatal and fatal overdose and for contracting HIV and HCV (which currently have a low prevalence in this sample) (Patel et al., 2024). Moreover, as described previously, Baltimore has a unique position in the overdose epidemic, increasing the relevance of our study population.

In 2024, there was a significant decrease in the number of fatal overdoses reported in Baltimore – marking a return to pre-pandemic levels (“Pages - MDH Interactive Dashboards,” 2025). Similar trends were seen across the US (Abuse, 2024). While these declines are worth noting, they were not equally distributed, and rates of overdose remain high. In this study, we saw that many ALIVE participants, particularly those recently recruited from the Baltimore community of people who have injected drugs, report using multiple substances, primarily fentanyl/heroin and cocaine but also xylazine, methamphetamine, and prescription drugs, as well as daily injection (the latter of which is consistent with prior cohorts at the time of their enrollment) (Cepeda et al., 2019). There are still many people at high risk for overdose and other substance use harms in Baltimore. As has been seen with the emergence of fentanyl and xylazine in the US, the drug market is ever in flux and can shift rapidly, which complicates efforts to combat the overdose epidemic (Cohn et al., 2025). Consequently, there must remain continued efforts to make effective harm reduction strategies and substance use treatment more accessible, to develop treatments for non-opioid substances (e.g., stimulants and xylazine), and to monitor changes in the drug supply as they occur.

## CRediT authorship contribution statement

**Liz M. Martinez Ocasio:** Writing – review & editing, Software, Investigation, Formal analysis. **Kenneth A. Feder:** Writing – review & editing, Conceptualization. **Jacqueline E. Rudolph:** Writing – original draft, Supervision, Software, Methodology, Investigation, Formal analysis, Conceptualization. **Catherine Tomko:** Writing – review & editing. **Javier A. Cepeda:** Writing – review & editing. **Gregory D. Kirk:** Writing – review & editing, Funding acquisition. **Becky L. Genberg:** Writing – review & editing, Supervision, Funding acquisition, Conceptualization. **Shruti H. Mehta:** Writing – review & editing, Funding acquisition. **Danielle German:** Writing – review & editing, Funding acquisition.

## Funding

This work was supported in part by 10.13039/100000002National Institutes of Health grants U01-DA036297 and R01-DA057673. This manuscript is the result of funding in whole or in part by the National Institutes of Health (NIH). It is subject to the NIH Public Access Policy. Through acceptance of this federal funding, NIH has been given a right to make this manuscript publicly available in PubMed Central upon the Official Date of Publication, as defined by NIH. The funders had no role in study design, data collection, data analysis, or preparation of the manuscript, or decision to publish.

## Declaration of Competing Interest

The authors declare the following financial interests/personal relationships which may be considered as potential competing interests: Jacqueline E Rudolph reports financial support was provided by National Institutes of Health. If there are other authors, they declare that they have no known competing financial interests or personal relationships that could have appeared to influence the work reported in this paper.
