# Supplementary material for: Characterizing patterns of opioid and stimulant use by route and associations with non-fatal overdose and xylazine use in people who have injected drugs from Baltimore, MD, 2023–2024
Source: Drug Alcohol Depend Rep. 2025 Dec 6;18:100403. doi: 10.1016/j.dadr.2025.100403 (PMC12757556; doi:10.1016/j.dadr.2025.100403)
Supplement: Supplementary file 1 — Supplementary material [file mmc1.docx]

**Appendix A. Details on Study Recruitment**

Since the inception of the AIDS Linked to the IntraVenous Experience study in 1988, there have been five additional recruitment waves: 1994-1995, 1998, 2005-2008, 2015-2018, and 2023-present. With each wave, the cohort is replenished with new members from the community, through a combination of referrals from current cohort participants and field-based recruitment at locations that people who use drugs frequent, such as homeless shelters, syringe service programs, medication for opioid use disorder (MOUD) programs, community clinics (including HIV clinics), and emergency rooms. Study staff and community organizations also perform street outreach. Individuals are eligible for study inclusion if they are over the age of 18 and have injected drugs in the previous 10 years (1988 recruitment cohort) or in the previous 5 years (2023 recruitment cohort). Participants receive monetary compensation at each follow-up study visit they attend ($35 in 1988; $57 in 2023), in addition to free and confidential HIV testing, post-HIV test counseling, education on HIV and HCV prevention, and referrals to medical and social services.

Appendix B. Supplementary table

Supplementary Table 1. Distribution of participant characteristics and experiences by enrollment cohort.

| Variable | Overall  (n=1132) | Pre-2005  (n=263) | 2005  (n=173) | 2015  (n=224) | 2023  (n=472) |
| --- | --- | --- | --- | --- | --- |
| Sociodemographic |  |  |  |  |  |
| Age, median (IQR) | 56.3 (44.1, 63.2) | 64.8 (60.9, 68.6) | 60.2 (55.2, 65.3) | 55.0 (49.2, 60.9) | 44.4 (37.6, 55.6) |
| Female sex | 377 (33%) | 81 (31%) | 66 (38%) | 74 (33%) | 156 (33%) |
| Race/ethnicity |  |  |  |  |  |
| Non-Hispanic Black | 760 (67%) | 256 (97%) | 145 (84%) | 148 (66%) | 211 (45%) |
| Non-Hispanic white | 306 (27%) | 0 (0%) | 20 (12%) | 63 (28%) | 223 (47%) |
| Hispanic | 9 (1%) | 4 (2%) | 1 (1%) | 3 (1%) | 1 (<1%) |
| Other | 57 (5%) | 3 (1%) | 7 (4%) | 10 (4%) | 37 (8%) |
| Never married | 622 (55%) | 137 (52%) | 84 (49%) | 133 (59%) | 268 (57%) |
| Less than high school | 537 (47%) | 150 (57%) | 101 (58%) | 117 (52%) | 169 (36%) |
| Employed | 115 (10%) | 49 (19%) | 14 (8%) | 29 (13%) | 23 (5%) |
| Any homelessness | 354 (31%) | 11 (4%) | 13 (8%) | 45 (20%) | 285 (60%) |
| Incarcerated ≥1 week | 40 (4%) | 2 (1%) | 2 (1%) | 7 (3%) | 29 (6%) |
| Residence |  |  |  |  |  |
| In City | 872 (77%) | 237 (90%) | 143 (83%) | 188 (84%) | 304 (64%) |
| Outside City | 177 (16%) | 20 (8%) | 24 (14%) | 29 (13%) | 104 (22%) |
| No geocoding | 83 (7%) | 6 (2%) | 6 (3%) | 7 (3%) | 64 (14%) |
| Substance use |  |  |  |  |  |
| Age at first injection, median (IQR) | 18 (14, 23) | 20 (17, 25) | 21 (17, 28) | 21 (18, 27) | 14 (12, 17) |
| Any injection use | 524 (46%) | 21 (8%) | 24 (14%) | 63 (28%) | 416 (88%) |
| Any non-injection use | 720 (64%) | 66 (25%) | 63 (36%) | 146 (65%) | 445 (94%) |
| Any MOUD | 559 (49%) | 80 (30%) | 91 (53%) | 125 (56%) | 263 (56%) |
| Drug support group | 385 (34%) | 64 (24%) | 66 (38%) | 76 (34%) | 179 (38%) |
| Overdose | 129 (11%) | 8 (3%) | 6 (3%) | 19 (9%) | 96 (20%) |
| Clinical |  |  |  |  |  |
| Depressive symptoms | 374 (33%) | 36 (14%) | 34 (20%) | 68 (30%) | 236 (50%) |
| HIV | 222 (20%) | 68 (26%) | 71 (41%) | 59 (26%) | 24 (5%) |
| HCV viremia | 775 (71%) | 221 (84%) | 139 (80%) | 163 (73%) | 252 (58%) |

Abbreviations: IQR, interquartile range; n, number of participants
